# Serological evidence of H9N2 avian influenza virus exposure among poultry workers from Fars province of Iran

**DOI:** 10.1186/s12985-016-0472-z

**Published:** 2016-01-27

**Authors:** A. Heidari, M. Mancin, H. Nili, G. H. Pourghanbari, K. B. Lankarani, S. Leardini, G. Cattoli, I. Monne, A. Piccirillo

**Affiliations:** Research and Innovation Department, Istituto Zooprofilattico Sperimentale delle Venezie, OIE/FAO and National Reference Laboratory for Newcastle Disease and Avian Influenza, OIE collaborating Center for Diseases at the Human-Animal Interface, Viale dell’Università 10, Legnaro, PD 35020 Italy; Department of Comparative Biomedicine and Food Science, University of Padua, Legnaro, PD Italy; Food safety department, Istituto Zooprofilattico Sperimentale delle Venezie (IZSVe), Viale dell’Università 10, Legnaro, PD 35020 Italy; Avian Diseases Research Center, School of Veterinary Medicine, Shiraz University, Shiraz, Iran; School of Veterinary Medicine, Ardakan University, Yazd, Iran; Health Policy Research Center of Shiraz University of Medical Science, Shiraz, Iran

**Keywords:** H9N2, Avian influenza, Iran, Poultry workers, Hemagglutination inhibition (HI), Microneutralization (MN)

## Abstract

**Background:**

Since the 1990s, influenza A viruses of the H9N2 subtype have been causing infections in the poultry population around the globe. This influenza subtype is widely circulating in poultry and human cases of AI H9N2 have been sporadically reported in countries where this virus is endemic in domestic birds. The wide circulation of H9N2 viruses throughout Europe and Asia along with their ability to cause direct infection in mammals and humans, raises public health concerns. H9N2 AI was reported for the first time in Iran in 1998 and at present it is endemic in poultry. This study was carried out to evaluate the exposure to H9N2 AI viruses among poultry workers from the Fars province.

**Methods:**

100 poultry workers and 100 healthy individuals with no professional exposure to poultry took part in this study. Serum samples were tested for antibodies against two distinct H9N2 avian influenza viruses, which showed different phylogenetic clustering and important molecular differences, such as at the amino acid (aa) position 226 (Q/L) (H3 numbering), using haemagglutination inhibition (HI) and microneutralization (MN) assays.

**Results:**

Results showed that 17 % of the poultry workers were positive for the A/chicken/Iran/10VIR/854-5/2008 virus in MN test and 12 % in HI test using the titer ≥40 as positive cut-off value. Only 2 % of the poultry workers were positive for the A/chicken/Iran/12VIR/9630/1998 virus. Seroprevalence of non exposed individuals for both H9N2 strains was below 3 % by both tests. Statistical analyses models showed that exposure to poultry significantly increases the risk of infection with H9N2 virus.

**Conclusions:**

The results have demonstrated that exposure to avian H9N2 viruses had occurred among poultry workers in the Fars province of Iran. Continuous surveillance programmes should be implemented to monitor the presence of avian influenza infections in humans and to evaluate their potential threat to poultry workers and public health.

**Electronic supplementary material:**

The online version of this article (doi:10.1186/s12985-016-0472-z) contains supplementary material, which is available to authorized users.

## Background

Most emerging diseases are of zoonotic origin, with wild and domestic animals acting as natural reservoirs [1]. Globalization and intensive animal farming have led to an increased spread of zoonotic infections [2]. Influenza type A viruses include several distinct subtypes based on the antigenic properties of the two major surface glycoproteins, the hemagglutinin (HA) and the neuraminidase (NA). To date, 18 subtypes of HA (H1-H18) and 11 subtypes of NA (N1-N11) have been described [3]. A number of influenza A subtypes have successfully crossed the species barrier and have established in the mammals and human population, causing yearly seasonal epidemics or they have sporadically been directly transmitted from poultry to humans causing zoonotic infections [4, 5]. The influenza A viruses of the H9N2 subtype are classified as low pathogenic avian influenza (LPAI) viruses. They cause infections both in wild birds and in the poultry population worldwide, including several countries in Asia, Europe, North Africa and North America [6, 7]. A significant proportion of recent H9N2 avian influenza (AI) isolates contains the L226Q (H3 numbering) amino acid substitution in their hemagglutinins (HAs) showing preferential binding to analogs of receptors with sialic acid linked to galactose by α2,6 linkage (SAα2,6Gal), a phenotypic portrait which is characteristic of human influenza viruses. Thus, these AI viruses might possess one of the key elements for infection in humans [8–10]. Indeed, H9N2 viruses were isolated for the first time from humans in Hong Kong in 1999 and further human infections were reported in 2003 [11, 12]. These studies have shown that avian H9N2 viruses isolated from chickens are closely related to the H9N2 viruses responsible for human infection [13]. One human case of H9N2 AI was reported in Bangladesh [14] and the World Health Organization (WHO) in 2015 has reported new cases in Egypt and Bangladesh [15, 16]. In 1998, domestic pigs from Hong Kong were confirmed as being infected with H9N2 influenza, and infections have been reported also in recent years in swine along with other mammals [17, 18]. Furthermore, H9N2 viruses can contribute with gene segments during reassortment events leading to the generation of novel avian influenza virus that can infect humans (e.g. recent Chinese H7N9 and H10N8 viruses) [19, 20]. Recent transmission studies have demonstrated that some natural isolates of H9N2 viruses can acquire the ability to transmit efficiently between ferrets via respiratory droplets. In addition, it has been reported that serial passages of an H9N2 virus through guinea pigs can result in the introduction of amino acid substitutions, which increases contact transmission efficiency in this mammalian model [21, 22].

The wide circulation of H9N2 viruses throughout Eurasia, along with their ability to cause direct infections in mammals and humans, raises public health concerns on their potential role as candidates for the next influenza pandemic [23]. H9N2 human infection is generally asymptomatic or responsible for mild clinical signs. This may explain the scarcity of evidence accounting for the circulation and transmission of this virus subtype [24]. Nonetheless, human sera positive for H9 subtype were identified in China, India, Iran, Thailand, Cambodia, Romania, Egypt and Pakistan [25–34].

In Iran, the H9N2 subtype was identified for the first time in 1998 and is still circulating in the poultry population. In the affected farms the mortality rate ranges between 20 and 60 %, although this may also be attributable to co-infections with other pathogens, such as IBV or Mycoplasma gallisepticum [35]. In spite of the implemented national control measures, which include the mass vaccination of poultry, the virus has rapidly spread and can be considered endemic in the Iranian poultry [36].

Exposure to H9N2 AI viruses in Iranian poultry workers was previously revealed by means of HI test, using serum titre **≥**20 as positive cut-off [28, 29]. In previous reported studies, the H9N2 AI seroprevalence, assessed by means of HI test, in Iranian poultry workers ranged from 1.6 to 15.7 % (Median 9.5). Amongst the Middle-Eastern and Southern Asia countries, the highest seroprevalence was observed in Pakistan (47.8 %) by means of HI test [34] and 7.5 % by means of MN test in Egypt [33]. In the present study, two different serological tests were used and compared to screen 200 individuals from the Fars province, Iran to better understand the risk of infection with the H9N2 virus in the poultry sector. The two different diagnostic tests were applied to assess whether (i) the human exposure to this virus subtype can be confirmed and whether (ii) the poultry operators in the Iranian endemic areas (the Fars province, in this study) are at risk of exposure to the H9N2 infection.

## Results

**Fig. 1:**
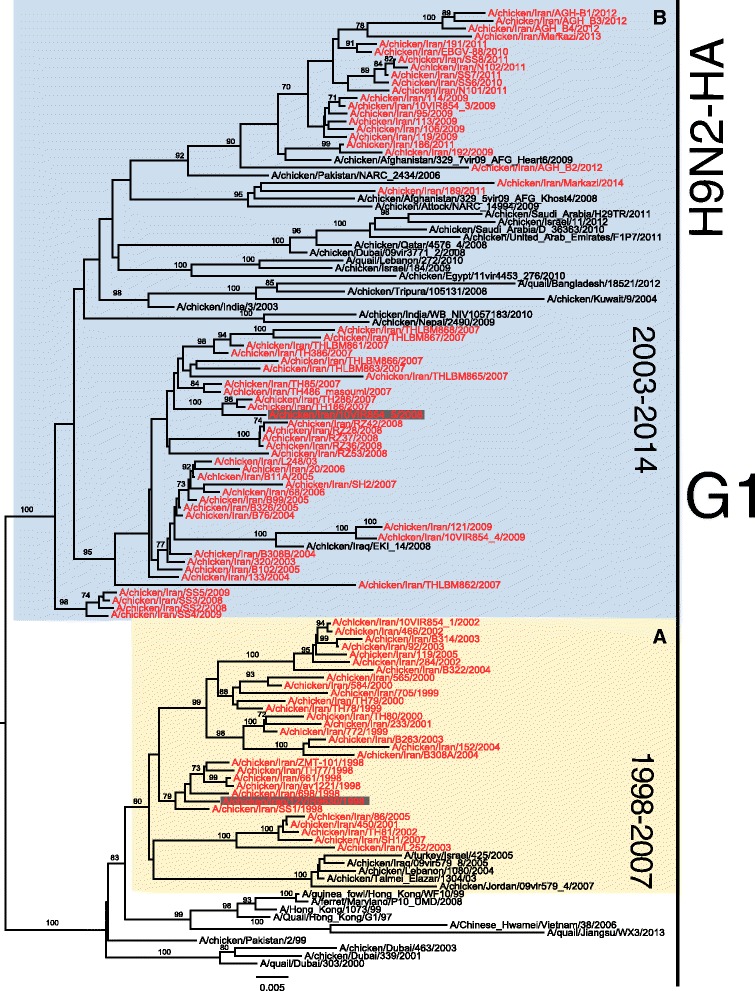
Neighbour-Joining nucleotide Phylogenetic tree with 1000 bootstrap of the AI H9N2 HA gene of the G1 lineage. Sub-lineages **a** and **b** are highlighted with yellow and blue frames respectively, Iranian Sequences are highlighted in red and two Iranian strains used in serological analyses are highlighted with gray square. Numbers at the nodes represent bootstrap values (≥70)

The HA gene phylogenetic analysis of 88 Iranian H9N2 strains collected between 1998-2014 shows that they belong to two different groups of G1 lineage, named sub-lineage A and sub-lineage B for the purpose of this study (Fig. 1). Sub-lineage A refers to the H9N2 isolates collected between 1998 and 2007, which apparently are no longer circulating among the Iranian poultry, while B includes more recent isolates collected between 2003 and 2014. The amino acid sequence analyses of the Iranian H9N2 viruses showed that sub-lineage A consists of 47 % (14/30) of the strains with amino acid Q at position 226 (H3 numbering) and 53 % (16/30) with L226 substitution. Interestingly, sub-lineage B encloses mostly (95 %) (55/58) the strains with amino acid L226. Based on these results, two Iranian H9N2 isolates representatives of sub-lineages A (A/chicken/Iran/12VIR/9630/1998) and B (A/chicken/Iran/10VIR/854-5/2008) were selected as antigens for the serological study (Fig. 1). In particular, A/chicken/Iran/12VIR/9630/1998 was characterized by the amino acid Q226 while the A/chicken/Iran/10VIR/854-5/2008 virus had amino acid L at this position. The HA amino acid sequence distance between the two selected strains was 8.1 %.

The percentage of seropositive individuals against different influenza viruses obtained with the HI and MN tests along with the p-value of the chi-square test related to the association between exposure and response to each virus tested, per method are reported in Table 1. Detailed results of serological investigations obtained for the exposed and unexposed groups against different influenza viruses are described in the supplementary material (Additional file 1: Table S2). Serological results showed that the prevalence of antibodies against A/chicken/Iran/12VIR/9630/1998 H9N2 (Q 226) was of 2 % in the exposed group by the HI and MN tests, while no positive results were identified in the unexposed group.

**Table 1:** Positive titer percentages and p-value of the chi square test related to the association between exposure and response to each virus tested, per method

	Triple test average (100 sample subjects)					
Virus	HI			MN		
	% Positive	% Positive	*p*-value	% Positive	% Positive	*p*-value
	Exposed group	Unexposed group		Exposed group	Unexposed group	
H9N2 A/chicken/Iran/12VIR/9630/1998	2	0	0.01	2	0	0.01
H9N2 A/chicken/Iran/10VIR/854-5/2008	12	2	0.005	17	3	0.001
H1N1pdm 2009 A/California/4/2009	28	53	0.0003	25	33	0.02
H3N2 A/Minnesota/11/2010	36	44	0.02	28	24	0.05

**Fig. 2:**
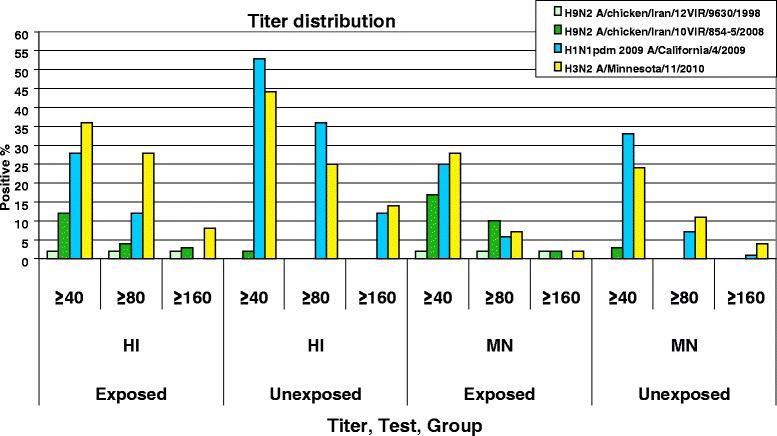
Graphic distribution of titer categories expressed in percentage among the subjects of the exposed and unexposed groups in the HI and MN tests

An association was observed between professional exposure to poultry species and the presence of antibodies to the A/chicken/Iran/10VIR/854-5/2008 H9N2 (L 226) virus, as revealed by the HI and MN test (*p* < 0.05). The percentage of positivity was of 12 % for the exposed group and 2 % for the unexposed group in the HI test, while it respectively amounted to 17 % and 3 % for the exposed and unexposed groups in the MN test. Antibody prevalence to H1N1pdm 2009 was of 28 % in the exposed versus 53 % in the unexposed groups by the HI test, and 25 % versus 33 %, respectively, by the MN test. For H3N2, the prevalence was 36 % in the exposed group compared to 44 % in the unexposed group with the HI test, while it was 28 % and 24 % in the exposed and unexposed groups with the MN test. The titer distribution percentage against different viruses in different tests (Fig. 2) showed that with the increase of the titre range from ≥40 to ≥80 and from ≥80 to ≥160, the percentage of antibody positivity drastically decreased. Cross MN and HI tests on homologues and different chicken H9N2 hyper immune sera used as positive controls showed the absence of cross reactivity with H1N1pdm 2009 and H3N2 seasonal viruses; the same results were observable in human immune sera for H1N1pdm 2009 and H3N2, which showed the absence of a cross reactivity with the two tested H9N2 viruses.

The probability to be positive against A/chicken/Iran/10VIR/854-5/2008 H9N2, H3N2 and H1N1pdm 2009 viruses in different tests was calculated. The generalized linear model for binary responses applied on the H9N2 viruses indicated that the association between professional exposure or absence of professional exposure and H9 infection was significant (*p* = 0.0018). In particular, the exposed and unexposed groups showed a significantly different positivity both in the HI test (9 % vs. 1 %) and in the MN test (13 % vs. 2 %). For the H3N2 virus, the professional exposure was not significantly associated to the presence of antibodies (*p* = 0.22). This means that in both groups the probability of testing positive is almost similar. Differently, the unexposed group showed a significantly higher number of individuals (*p* = 0.001) seropositive to H1N1pdm 2009. If we consider that the H1N1pdm 2009 subtype does not circulate in avian species, it can be assumed that the serological data obtained for this subtype do not depend on the exposure to poultry species; other factors may have been at the origin of the distinct immunoreactivity observed against the H1N1pdm 2009 subtype in the two groups under study.

## Discussion and Conclusion

Results demonstrated that exposure to avian H9N2 viruses had occurred in humans in Iran (Fars province). In particular, the analysis showed a significantly higher prevalence of neutralizing antibodies against the A/chicken/Iran/10VIR/854-5 H9N2 virus in poultry workers than in the professionally unexposed group. These results are in accordance with a previously reported study from Iran by Alizadeh et al. [28] confirming that poultry workers are at risk of infection from zoonotic avian influenza virus. For the first time in this study, two distinct H9N2 LPAI viruses were used as antigens for serological investigations in humans. Interestingly, neutralizing activity against just one of these two antigens was revealed in several sera, which has resulted in significant different prevalence. This is consistent with the amino acid diversity of the two H9N2 antigens used in this study. In fact, the HA amino acid sequences of A/chicken/Iran/12VIR/9630/1998 (Q-226) and A/chicken/Iran/10VIR/854-5/2008 (L-226) differed for 43 amino acids, seven of which were located in antigenic sites (S158N, S160L, S175D, E190A, S193N, N208D, Q226L (H3 numbering)) previously reported by other groups [37–40]. These amino acid substitutions may account for the different serological reactivity against these antigens. However, the difference in prevalence observed in this study between two tested H9N2 viruses might have different explanations.

The receptor binding properties of A/chicken/Iran/12VIR/9630/1998 H9N2 (Q-226)-like viruses affect their ability to infect humans. As a consequence, the infections of this virus in the exposed population are limited. On the contrary, the A/chicken/Iran/10VIR/854-5 H9N2 (L-226) – like viruses, have increased ability to infect humans resulting in higher prevalence in the exposed population. However, it should be considered that the molecular analysis of Iranian H9N2 viruses demonstrated that those harbouring the Q-226 mutations apparently are no longer in circulation. Furthermore, one previous study investigating the serologic response in humans exposed to avian viruses concluded that the incidence of seroconversion is low and that the antibody response after mild/asymptomatic infections is short-lived [41]. Therefore, the reduction of the circulation of Q-226 viruses in the poultry in recent times and/or the low antibody response after infection might be responsible for the decreased prevalence of antibodies against these AI viruses.

Consistently with the results discussed so far, the unexposed group counting 100 healthy subjects showed a very low percentage of positivity for H9N2 by the HI and MN tests, while the distribution of positive titres against H1N1pdm 2009 and seasonal H3N2 influenzas were similar in the exposed and in the unexposed groups.

According to Stephenson et al. [42] the cross reacting antibodies against H2 might explain the antibody reactivity against avian H9N2, especially among people born before 1968. Although we did not test the sera for this antigen, to evaluate this hypothesis, the age of each individual who had tested H9 positive was verified. Only one sample was from a person born before 1968, while all the others were collected from younger subjects with an age between 22-46 years, who could not be positive for H2N2.

The comparison between the serological results obtained using the HI and MN tests against H9N2 showed that the MN test detected more positive samples within the same group, however, the triple test results proved that the HI titer was reasonably more consistent than the MN test. In addition, different researches have reported that intra-laboratory reproducibility by MN is lower than the one resulting from the HI test [43], a fact that may explain the moderate discrepancy observed between the two serological assays applied for the detection of antibodies against H1N1pdm 2009 and H3N2 seasonal influenza. Both in the HI and MN tests, the percentage of positivity for H9N2 decreased with the increase of the titre range; the obtained results were justified on the grounds that humans have a poor immune response to infections with avian influenza viruses. For instance, studies have shown that the individuals with mild or asymptotic infections had a lower antibody titer compared to individuals who had seriously developed the illness [44]. H9N2 AI viruses have some of the specific molecular markers for the infection of mammals; however, they are still unable to completely adapt to mammals and to cause serious disease in humans, which may explain the low titers observed. As well, the time elapsed between the infection and sampling dates can also account for the decrease of the antibody titer.

In conclusion, this study has highlighted the potential of avian to human transmission of H9N2 AIVs, and indicated that poultry workers are at risk of infection. Avian transmission of H9N2 viruses in humans can increase the probability of human adaption, while genetic reassortment with other human seasonal viruses are means of generating an influenza virus with epidemic or pandemic potential. Hence, integrated medical-veterinary surveillance and research activities are essential in order to identify the emergence of new influenza viruses, assess the clinical significance of seropositivity and understand more on the mechanisms that favour the virus to cross the species barrier. In the poultry sector, surveillance and control programmes should be implemented to reduce the prevalence of H9N2 AIV in poultry population and minimize the risk of exposure in poultry operators.

## Methods

### Study population and sample collection

The analyses were conducted on the blood sera of 100 workers regularly exposed to poultry (exposed group) and of 100 individuals with no professional exposure to poultry (unexposed group). The individuals, belonging to the exposed group, were recruited among the operators of the poultry industry and of the University Poultry Veterinary Hospital, none of whom had been vaccinated against flu. In particular, blood samples were collected between September – December 2012 from 70 slaughterhouse workers, 30 poultry house workers and from 10 operators of the University Poultry Veterinary Hospital, all resident in the Fars province (southern Iran). In January 2013, 100 sera were also collected from subjects from the same province not professionally exposed to poultry. 98 % of the individuals included in the study were males, whose mean age among the poultry workers was of 32 years (ranging between 18–62). The mean age in the professionally unexposed group was of 41 years (range between 18–67). After blood collection the serum was separated, stored at –20 °C and subsequently sent to IZSVe for the serological analyses.

### Ethics statement

The use of serum samples for research purposes was approved by the Veterinary University of Shiraz in compliance with the Iranian ethical principles and institutions and according to the principles expressed in the World Medical Association Declaration of Helsinki. Informed consent was obtained from all participating individuals.

### Selection of antigens and sera

To select representative H9 antigens to be used in the serological investigations, the HA gene nucleotide and amino acid sequences of five Iranian H9N2 AIV isolates available at IZSVe repository were analysed and compared with the Iranian sequences available in the GenBank. Subsequently, a nucleotide sequence dataset of 88 Iranian strains along with representative strains of G1 lineage were aligned and phylogenetically analysed constructing a Neighbour-Joining phylogenetic tree by the MEGA 5 (http://www.megasoftware.net) program (see results). In addition, sera of all individuals were tested for the presence of antibodies to seasonal A/Minnesota/11/2010 H3N2 and A/California/4/2009 H1N1pdm 2009 viruses. Positive control sera containing antibodies directed against the selected H9N2 antigens were produced in SPF chickens. In addition, a panel of different H9N2, H1N1, H3N2 chicken sera and an H1N1, H3N2 positive human sera were used as controls in the serological assays.

### Serological methods

#### Hemagglutinin inhibition (HI) test

The HI assay was applied according to the World Health Organization (WHO) and World Organisation for Animal Health (OIE) manual [45, 46]. Briefly, 0.5 % (vol/vol) chicken red blood cell (RBC) solution was prepared by washing with Phosphate buffered saline (PBS) containing 0.5 % Bovine serum albumin (BSA) (Sigma). To remove non specific serum inhibitors, the human serum samples were treated with RDE (receptor destroying enzyme) (Sigma-Aldrich) that was reconstituted with 5 ml sterile distilled water. 50 μl of serum was added to 200 μl of RDE diluted to 100 ml with calcium saline, PH 7.2 and incubated overnight at 37 °C. Then 5 vol of 1.5 % sodium citrate were added then heated at 56 °C for 30 min to inactivate remaining RDE. The treated sera were tested by haemagglutination assay to verify the presence of non-specific agglutination. After that the treated serum (50 μl of each one) was diluted in two-fold serial dilutions (1:10) with 25 μl PBS in 96-well V-bottom microtiter plates. Subsequently, 25 μl of virus antigens containing 4 HA units were added to the wells and incubated at room temperature (RT) for 30–45 min. 50 μl of 0.5 % chicken RBCs were then added and the plate was incubated further for 45 min at RT before recording the agglutination titers. The HI test results were expressed as the reciprocal of the last dilution of the sample that completely inhibited haemagglutination. In all the assays control positive serum samples were included, and all assays were tested in triplicate.

#### Microneutralization (MN) test

The WHO MN test protocol was applied [47]. The human serum samples were inactivated by heating at 56 °C for 30 min H9N2, H3N2 and H1N1pdm virus stocks used in our analyses were titrated in the presence of TPCK-trypsin (2 μg/ml) (Sigma-Aldrich) for determination of tissue culture infectious dose (TCID50). 10 μl of treated sera were added to 96 well cell culture plates (Costar) and 2-fold serial dilutions were performed. Inoculum was prepared in virus diluent with the addition of TPCK-trypsin (2 μg/ml) so that 50 μl contained 100 TCID50 of the respective virus. Fifty microliters of virus inoculum was added to the wells, the virus serum mixture was subsequently incubated for 1 hr at 37 °C, 5 % CO2. A back-titration of the virus inoculum was performed in each assay using 2 fold serial dilutions. 100 μl MDCK cells (1.5 × 104 cells /well) were then added to each well and the plate was incubated overnight at 37 °C, 5 % CO2 (18–20 hrs). The plate was then fixed with 100 μl/well of cold fixative (80 % Acetone in PBS) for 10 min. The virus was detected with an anti-Influenza A, Nucleoprotein monoclonal antibody (Merck Millipore) and Peroxidase conjugated-goat anti-mouse IgG (γ) (KPL) as secondary antibody using ELISA. All serum samples and controls were tested in triplicate.

#### Criteria for seropositivity

The WHO guidelines for vaccine evaluation suggest that a neutralizing antibody titre ≥40 indicate higher than 50 % protection against influenza A virus infection or disease. Based on this consideration, an individual with an antibody titer ≥40 was considered positive for different serotype in MN and HI tests [41].

#### Statistical Analyses

The Geometric Mean titers (GMTs) of the triple test per method (HI and MN) was calculated for each subject checked for the virus of interest. A binary variable was created to identify the positive/negative sample. An average titre lower than 40 identified negative samples, whereas an average titre higher than 40 identified positive samples. The Chi square test was used to verify the possible association between positivity and exposure, for each method and virus. The GLM (Generalized linear model) for binary responses was used to estimate the probability to have a positive reaction for each tested virus, considering simultaneously the methods (HI and MN), the groups (exposed and unexposed) and their interaction as variables. The Maximum Likelihood method was used to estimate the parameters of the model. The Type III F-tests were applied to evaluate the overall effect of specified variables in the model. *P*-values lower than 0.10 were considered as significant [48]. SAS 9.3 software was used to fit the statistical analysis.

## Additional file

Additional file 1: Table S2. Results of serological investigations obtained for the different categories of exposed and unexposed groups against different influenza viruses. (DOC 110 kb)

## Abbreviations

aa: amino acid
AI: Avian Influenza
GMTs: geometic mean titers
HA: hemagglutinin
HI: hemagglutinin inhibition
MN: microneutralization assay
MDCK: Madin-Darby canine kidney
OIE: Organisation for Animal Health
pdm: pandemic
WHO: World Health Organization
