# Supplementary material for: Serological evidence of H9N2 avian influenza virus exposure among poultry workers from Fars province of Iran
Source: Virol J. 2016 Jan 27;13:16. doi: 10.1186/s12985-016-0472-z (PMC4728806; doi:10.1186/s12985-016-0472-z)
Supplement: Additional file 1: Table S2. — Results of serological investigations obtained for the different categories of exposed and unexposed groups against different influenza viruses. (DOC 110 kb) [file 12985_2016_472_MOESM1_ESM.doc]

**Additional file 1: Table S2. Results of serological investigations obtained for the different categories of exposed and unexposed groups against different influenza viruses.**

| **Virus, Age group, Work group** | **Subjects** | | | **HI** | | | **MN** | | |
| --- | --- | --- | --- | --- | --- | --- | --- | --- | --- |
|  | **Exposed subjects**  **NO.** | **Unexposed subjects**  **NO.** | | **Positivity % in Exposed (subjects NO.)** | **Positivity % in Unexposed (subjects NO.)** | **p-value** | **Positivity % in Exposed (subjects NO.)** | **Positivity % in Unexposed (subjects NO.)** | **p-value** |
|  |  |  |  |  |  |  |  |  |  |
| **H9N2**  **A/chicken/Iran/12VIR/9630/1998** |  | |  |  |  |  |  |  |  |
| Positive % |  | |  |  |  |  |  |  |  |
| Age group (Years) |  | |  |  |  |  |  |  |  |
| 18-39 | 80 | | 50 | 1 (1) | 0 | NS | 1 (1) | 0 | NS |
| 40-60 | 20 | | 50 | 5 (1) | 0 | 0.1 | 5 (1) | 0 | 0.1 |
| **Work category** |  | |  |  |  |  |  |  |  |
| Poultry House Worker | 20 | | - | 0 | NO | NO | 0 | NO | NO |
| Slaughterhouse Worker | 70 | | - | 1.43 (1) | NO | NO | 1.43 (1) | NO | NO |
| Vet Student in poultry Hospital | 10 | | - | 10 (1) | NO | NO | 10 (1) | NO | NO |
| Positive% ≥40 | 100 | | 100 | 2 (100) | 0 (100) |  | 2 (100) | 0 (100) |  |
| Positive% ≥80 | 100 | | 100 | 2 (100) | 0 (100) |  | 2 (100) | 0 (100) |  |
| Positive % ≥160 | 100 | | 100 | 2 (100) | 0 (100) |  | 2 (100) | 0 (100) |  |
| **TOT Positive %** | 100 | | 100 | 2 (100) | 0 (100) | NS | 2 (100) | 0 (100) | NS |
| **H9N2**  **A/chicken/Iran/10VIR/854-5/2008** |  | |  |  |  |  |  |  |  |
| Positive % |  | |  |  |  |  |  |  |  |
| Age (Years) |  | |  |  |  |  |  |  |  |
| 18-39 | 80 | | 50 | 13.7 (11) | 2(1) | 0.02 | 17.5 (14) | 2 (1) | 0.007 |
| 40-60 | 20 | | 50 | 5 (1) | 2(1) | NS | 15 (3) | 4 (2) | NS |
| **Work category** |  | |  |  |  |  |  |  |  |
| Poultry House Worker | 20 | | - | 5 (1) | NO | NO | 15 (3) | NO | NO |
| Slaughterhouse Worker | 70 | | - | 14.2 (10) | NO | NO | 17 (12) | NO | NO |
| Vet Student in poultry Hospital | 10 | | - | 10 (1) | NO | NO | 20 (2) | NO | NO |
| Positive% ≥40 | 100 | | 100 | 12 (100) | 2 (100) |  | 17 (100) | 3 (100) |  |
| Positive% ≥80 | 100 | | 100 | 4 (100) | 0 (100) |  | 10 (100) | 0 (100) |  |
| Positive % ≥160 | 100 | | 100 | 3 (100) | 0 (100) |  | 2 (100) | 0 (100) |  |
| **TOT Positive %** | 100 | | 100 | 12 (100) | 2 (100) | 0.005 | 17 (100) | 3 (100) | 0.001 |
| **H1N1pdm 2009**  **A/California/4/2009** |  | |  |  |  |  |  |  |  |
| Positive % |  | |  |  |  |  |  |  |  |
| Age (Years) |  | |  |  |  |  |  |  |  |
| 18-39 | 80 | | 50 | 35 (28) | 48(24) | NS | 30 (24) | 28(14) | NS |
| 40-60 | 20 | | 50 | 0 (0) | 58(29) | <0.0001 | 5 (1) | 38(19) | 0.005 |
| **Work category** |  | |  |  |  |  |  |  |  |
| Poultry House Worker | 20 | | - | 40 (8) | NO | NO | 40 (8) | NO | NO |
| Slaughterhouse Worker | 70 | | - | 27 (19) | NO | NO | 23 (16) | NO | NO |
| Vet Student in poultry Hospital | 10 | | - | 10 (1) | NO | NO | 10 (1) | NO | NO |
| Positive% ≥40 | 100 | | 100 | 28 (100) | 53 (100) |  | 25 (100) | 33 (100) |  |
| Positive% ≥80 | 100 | | 100 | 1 (100) | 36 (100) |  | 6 (100) | 7 (100) |  |
| Positive % ≥160 | 100 | | 100 | 0 (100) | 12 (100) |  | 0 (100) | 1 (100) |  |
| **TOT Positive %** | 100 | | 100 | 28 (100) | 53 (100) | 0.0003 | 25 (100) | 33 (100) | NS |
| **H3N2**  **A/Minnesota/11/2010** |  | |  |  |  |  |  |  |  |
| Positive % |  | |  |  |  |  |  |  |  |
| Age (Years) |  | |  |  |  |  |  |  |  |
| 18-39 | 80 | | 50 | 41 (33) | 46 (23) | NS | 32 (26) | 24 (12) | NS |
| 40-60 | 20 | | 50 | 15 (3) | 42 (21) | 0.03 | 10 (2) | 24 (12) | NS |
| **Work category** |  | |  |  |  |  |  |  |  |
| Poultry House Worker | 20 | | - | 40 (8) | NO | NO | 20 (4) | NO | NO |
| Slaughterhouse Worker | 70 | | - | 35 (25) | NO | NO | 32 (23) | NO | NO |
| Vet Student in poultry Hospital | 10 | | - | 30 (3) | NO | NO | 10 (1) | NO | NO |
| Positive% ≥40 | 100 | | 100 | 36 (100) | 44 (100) |  | 28 (100) | 24 (100) |  |
| Positive% ≥80 | 100 | | 100 | 28 (100) | 25 (100) |  | 7 (100) | 11 (100) |  |
| Positive % ≥160 | 100 | | 100 | 8 (100) | 14 (100) |  | 2 (100) | 4 (100) |  |
| **TOT Positive %** | 100 | | 100 | 36 (100) | 44 (100) | NS | 28 (100) | 24 (100) | NS |

NS: not significant

NO: No data
